# Time-Dependent Indirect Antioxidative Effects of Oat Beta-Glucans on Peripheral Blood Parameters in the Animal Model of Colon Inflammation

**DOI:** 10.3390/antiox9050375

**Published:** 2020-04-30

**Authors:** Łukasz Kopiasz, Katarzyna Dziendzikowska, Małgorzata Gajewska, Jacek Wilczak, Joanna Harasym, Ewa Żyła, Dariusz Kamola, Michał Oczkowski, Tomasz Królikowski, Joanna Gromadzka-Ostrowska

**Affiliations:** 1 Department of Dietetics, Institute of Human Nutrition Sciences, Warsaw University of Life Sciences, Nowoursynowska 159c, 02-776 Warsaw, Poland; lukasz_kopiasz@sggw.edu.pl (Ł.K.); ewa_zyla@sggw.edu.pl (E.Ż.); michal_oczkowski@sggw.edu.pl (M.O.); tomasz_krolikowski@sggw.edu.pl (T.K.); joanna_gromadzka_ostrowska@sggw.edu.pl (J.G.-O.); 2 Department of Physiological Sciences, Institute of Veterinary Medicine, Warsaw University of Life Sciences, Nowoursynowska 159, 02-776 Warsaw, Poland; malgorzata_gajewska@sggw.edu.pl (M.G.); jacek_wilczak@sggw.edu.pl (J.W.); dariuszkamola@gmail.com (D.K.); 3 Adaptive Food Systems Accelerator—Science Centre, Wrocław University of Economics and Business, Komandorska 118/120, 53-345 Wrocław, Poland; joanna.harasym@ue.wroc.pl; 4 Department of Biotechnology and Food Analysis, Wrocław University of Economics and Business, Komandorska 118/120, 53-345 Wrocław, Poland

**Keywords:** oat beta-glucan, peripheral blood, *colitis*, oxidative stress, rats

## Abstract

*Background*: Oat beta-glucans are polysaccharides, belonging to soluble fiber fraction, that show a wide spectrum of biological activity. The aim of this study was to evaluate the time-dependent antioxidative effect of chemically pure oat beta-glucan fractions, characterized by different molar mass, which were fed to animals with early stage of 2,4,6-trinitrobenzene sulfonic acid (TNBS) - induced *colitis*. *Methods*: The study was conducted on 150 adult male Sprague Dawley rats assigned to two groups—healthy control (H) and *colitis* (C) with colon inflammation induced by *per rectum* administration of TNBS. The animals from both groups were divided into 3 nutritional subgroups, receiving for 3, 7 or 21 days AIN-93M feed without beta-glucan (βG−) or with 1% (*w*/*w*) low molar mass oat beta-glucan (βGl+) or 1% (*w*/*w*) high molar mass oat beta-glucan (βGh+). After 3, 7 and 21 days, the animals were euthanized, peripheral blood was collected from the heart for further analysis. *Results*: The results of analyses performed on blood samples showed small changes in lymphocytes count and red blood cell parameters such as the number of red blood cell, mean corpuscular hemoglobin concentration and mean corpuscular volume (RBC, MCHC, MCV respectively) as well as normalization of antioxidant potential accompanying moderate inflammatory state of colon mucosa and submucosa. *Conclusion*: Oat beta-glucans exert an indirect antioxidant effect in animals with TNBS-induced *colitis*, with greater effectiveness in removing systemic effects of colon inflammation found for low molar mass oat beta-glucan.

## 1. Introduction

Non-specific chronic inflammatory bowel disease (IBD) is a multifactorial disease of unexplained etiology, with a diverse course and clinical symptoms, often also systemic ones. IBD mainly includes Crohn’s disease (CD) and ulcerative colitis (UC) [1]. These are chronic diseases, often starting at an early age, characterized by a long pre-duration with alternating periods of recurrence and remission. Epidemiological data indicate an increase in the frequency of IBD worldwide, with the highest number of CD and UC cases being diagnosed in northern Europe, Great Britain and North America [2]. The IBD pathomechanism is complex and multifactorial.

The etiology of IBD emphasizes complex interactions between genetic factors, changes in microbiota composition (dysbiosis) and environmental factors, which result in disorders of intestinal immune mechanisms [3,4]. Studies indicate that two interrelated mechanisms are important in the development of IBD—generating free radicals and proinflammatory—activating the cascade of immune response. In many patients in the course of IBD development, the systemic inflammatory response, which is associated with an increased production and release of proinflammatory cytokines and chemokines, is also accompanied by an increased production of reactive oxygen species (ROS) and reactive nitrogen species (RNS) [5]. In the active phase of the disease, increased levels of pro-inflammatory cytokines and reactive oxygen species are observed both in the intestinal wall and in peripheral blood and other organs [6,7]. Reactive oxygen species playing immunoregulatory functions in IBD patients may contribute to the initiation and/or progression of disease [8]. The overproduction of ROS associated with increased activation of immune cells induces a pathogenic cascade, which consequently intensifies inflammation and leads to oxidative damage to DNA, proteins and lipids [9]. Therefore, it is important to identify oxidative stress markers sensitive to therapeutic factors and supporting IBD therapy, which may not only have local effects on the gut-associated lymphoid tissue but may also have systemic effects. An important element of IBD treatment is pharmacotherapy, which is accompanied by modification of the diet. The beneficial dietary factors recommended in IBD diet therapy include soluble fractions of dietary fiber, which include among others cereal beta-glucans [10].

Beta-glucans are a group of polysaccharides composed of D-glucose molecules which are a component of the cell wall of the aleurone layer and cereal endosperm. Oat beta-glucans are linear glucose polymers connected by β-1,3 and β-1,4 glycosidic bonds, thanks to which they dissolve well in water [11,12,13]. Due to their specific structure, beta-glucans have a number of functional and pro-health properties. The results of studies indicate beneficial metabolic and clinical effects of beta-glucans, for example, reduction of the effects of dysfunction and/or loss of protective barrier of gastrointestinal mucosa. The biological activity of beta-glucans results from their ability to bind to the receptors of immune cells and thus modulate the immune response. The anti-inflammatory activity of beta-glucans is manifested by changes in the synthesis and secretion of pro- and anti-inflammatory cytokines [14]. In vivo experiments on animal models demonstrated that oat beta-glucans have the ability to stimulate the cells of the intestinal lymphoid tissue and exert anti-inflammatory and/or antioxidative response [15,16,17,18]. The effects of beta-glucans on the biochemical, hematological and redox balance parameters and antioxidant defense markers in peripheral blood in various stages of the disease are not yet known. Therefore, the aim of this study was to determine the effect of dietary supplementation with 1% of low and high molar mass oat beta-glucans on hematological and biochemical parameters of blood in rats with TNBS-induced *colitis* and to assess the ability of beta-glucans to modulate the peripheral immune and oxidative response in this animal model of experimentally induced IBD. The study was conducted in a time-dependent manner in order to investigate the gradual changes in the organism after TNBS administration and analyze the effects of dietary supplementation with beta-glucans at different stages of IBD development.

## 2. Materials and Methods

### 2.1. Antioxidant Capacity and Polyphenols Content in Feed with Beta-Glucan Fractions

Total polyphenol content (TPC) was measured using standard TPC assay with Folin-Ciocalteu reagent. 500.00 ± 0.01 mg of sample was weighed and extracted with 4 mL of solvent mixture (HCl con.: methanol: H_2_O in a proportion of 1:80:10 *v*/*v*/*v*). After 1 h extraction at room temperature (RT) the centrifugation was proceeded for 10 min with 3500× *g* (MPW-351, Poland). Twenty μL of each sample was taken and treated with standard TPC procedure. Measurements were taken spectrophotometrically at 765 nm. The final polyphenols content was recalculated for 1g of dry matter basis (d.b.) expressed as gallic acid equivalent. The same extract was used for antioxidant capacity measurement with 2,2-diphenyl-1-picrylhydrazyl radical. The antioxidant capacity was expressed as Trolox equivalent for 1 g of d.b.

### 2.2. Animals and Experimental Design

**Figure 1:**
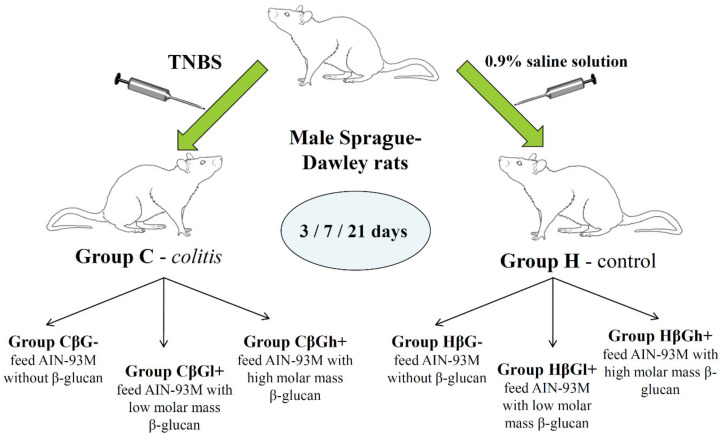
Scheme of experimental design of the study. TNBS: 2,4,6,6-trinitrobenzenesulfonic acid alcohol solution. Nutritional subgroups—AIN-93M with 1% (*w*/*w*) low molar mass oat beta-glucan (CβGl+; HβGl+;), AIN-93M with 1% (*w*/*w*) high molar mass oat beta-glucan (CβGh+; HβGh+) and AIN-93M without beta-glucan (CβG−; HβG−).

The experiment was performed on adult male Sprague-Dawley rats (*n* = 150) purchased from Charles River Laboratories (Sulzfeld, Germany). After one week of acclimatization to the animal house conditions (temperature 22 ± 1 °C, relative humidity 50 ± 5%, light/dark cycle 12/12 h, air exchange 15/hour, individual cages), the rats were divided into two groups—an experimental (*colitis* group—C) with *colitis* induced after rectal administration of 2,4,6,6-trinitrobenzenesulfonic acid alcohol solution (TNBS) and a control, healthy group (H), which was given 0.9% NaCl the same way. Animals in both groups were then divided into 3 nutritional subgroups, each of which received for 3, 7 or 21 days a different feed composition—AIN-93M with 1% (*w*/*w*) low molar mass oat beta-glucan (βGl+), AIN-93M with 1% (*w*/*w*) high molar mass oat beta-glucan (βGh+) and AIN-93M without beta-glucan (βG−). This formed 18 groups of 8 or 9 rats each (Figure 1). Feed intake was controlled every 2 days by calculating the standardized intake (g/day/100 g rat body weight). Body weight gain was measured every 7 days; therefore this measurement did not include groups fed specific diets for 3 days. The detailed description of the method of beta-glucans extraction from oats as well as the composition of the three types of animal feed used in this study are described in our previous article [18]. A schematic description of the stages of obtaining beta-glucans and their chemical structure is given in Supplementary Materials Figure S1.

After 3, 7 or 21 days of feeding with experimental feed or control feed, the rats in deep isoflurane anesthesia were bleed from the heart. All procedures were approved by the II Local Ethical Committee in Warsaw (Resolution No. 60/2015) in accordance with the Polish law and 3R principles (*Replacement, Reduction and Refinement*).

### 2.3. Blood Collection and Hematological Analysis

The peripheral blood collected from the heart was separated into two portions—whole blood and blood for plasma. In the part of whole blood, hematological analysis was performed using Abacus Junior Vet analyzer (BioMaxima, Lublin, Poland) and cytometric analysis was done using flow cytometer. Whole blood samples intended for lysis were frozen in liquid nitrogen. Plasma was obtained by centrifugation of whole blood at 2200× *g* for 10 min and then frozen in liquid nitrogen. Frozen plasma and whole blood samples were stored at −80 °C until biochemical analyses were performed. Hematological analysis determined the total number of white blood cells (WBC), monocytes and eosinophils (MID), lymphocytes (LYM), granulocytes (GRA), red blood cells (RBC) and platelets (PLT), as well as the mean corpuscular volume (MCV) and red cell distribution width (RDW) and the mean corpuscular hemoglobin concentration (MCHC) and mean corpuscular hemoglobin (MCH) in the cells as well as hemoglobin concentration (HGB) and hematocrit (HCT) of blood.

### 2.4. Flow Cytometric Analysis

Peripheral blood mononuclear cells (PBMCs) were isolated from the whole blood according to the method described previously [19]. Briefly, PMBCs were isolated by density gradient centrifugation in Histopaque-1077 (Sigma Aldrich, St. Louis, MO, USA) and then stained with antibodies against selected surface markers characteristic for specific subpopulations of lymphocytes (lymphocyte T, B and natural killer cells (NK), using commercially available sets of antibodies—Rat T Lymphocyte Cocktail (BD Biosciences, San Jose, CA, USA, cat. no: 558493) and Rat T/B/NK Cell Cocktail (BD Biosciences, San Jose, CA, USA, cat. no: 558495). The stained cells were counted using the FACSAria™ II flow cytometer (BD Biosciences, San Jose, CA, USA). The population of lymphocytes was first gated based on morphological characteristics—forward scatter (FSC) and side scatter (SSC) (gate P1). Then, cells located in gate P1 were analyzed with regard to their positive staining with appropriate antibodies. The results were expressed as a percentage of cells within the gated area (P1).

### 2.5. Biochemical Analysis

#### 2.5.1. Plasma Activity of Aminotransferases and Alkaline Phosphatase

The plasma activity of alanine aminotransferase (ALT), aspartate aminotransferase (AST) and alkaline phosphatase (ALP) was determined using ready-made analytical kits (Human Gesellschaft für Biochemica and Diagnostica mbH, Wiesbaden, Germany) based on kinetic methods recommended by the Expert Panel of the IFCC (International Federation of Clinical Chemistry, Milano, Italy).

#### 2.5.2. Activity of Antioxidant Enzymes and Glutathione Concentration

Blood cell lysate used for determination of antioxidant enzymes activity was prepared according to the procedure described in the instructions included in the kit for determination of glutathione reductase by Randox (County Antrim, United Kingdom).

The activity of superoxide dismutase (SOD), glutathione peroxidase (GPx) and reductase (GR) was determined using reagent kits from Randox (County Antrim, United Kingdom). Catalase activity (CAT) was determined in blood lysate according to the procedure described by Wheeler et al. [20]. The absorbance was measured spectrophotometrically at 550 nm light wavelength. The total concentration of glutathione (GSH) and glutathione disulfide (GSSG) was determined calorimetrically using the Glutathione Assay Kit from Cayman Chemical (Ann Arbor, MI, USA) according to the attached instruction manual.

#### 2.5.3. Plasma Total Antioxidant Status and Oxidative Stress Parameters

Total Antioxidant Status (TAS) in plasma was determined using Total Antioxidant Status Kit (Randox, County Antrim, United Kingdom). Lipid peroxidation level was determined as the concentration of thiobarbituric acid reactive substances (TBARS) according to Ohkawa et al. [21]. The concentration of TBARS expressed as malonic aldehyde (MDA) was calculated from the standard curve.

### 2.6. Statistical Analysis

The data obtained were analyzed using Statistica software version 13.1 PL (StatSoft, Cracow, Poland). Normality of distribution and equality of variance were determined for all data. Statistical analysis was carried out in two stages. In the first stage, separately for each time point (3, 7 and 21 days), two-way analysis of variance—ANOVA (colon inflammation vs. dietary intervention) was performed. In the second stage, a three-way analysis of variance—ANOVA (colon inflammation vs. dietary intervention vs. period of its use) was performed for all time points. The significance of differences between the groups was determined by the *post-hoc* Tukey test. Moreover, the results from all nutritional subgroups were compared with the control subgroup (HβG−) by Dunnett’s *post-hoc* test. The differences were considered statistically significant at *p* < 0.05. To assess the time-dependent effect between experimental factors, Fischer’s Linear Discriminant Analysis (LDA) was conducted. This method allows finding linear combinations of analyzed parameters (qualified as Fisher linear discriminators—FLD1 and FLD2) that separate the experimental groups of animals at three selected time points in the present study (3, 7 and 21 days).

## 3. Results

### 3.1. Antioxidant Potential of Feed and Oat Beta-Glucans

We suspected that antioxidant action observed as the result of the digestion of feeds supplemented with beta-glucan is not connected with any polyphenols that were present in the feed or their antioxidant activity. For that concept, the total polyphenols content and antioxidant activity vs. 2,2-diphenyl-1-picrylhydrazyl (DPPH) were assessed. Polyphenols in feed and beta-glucan fractions were estimated using Folin-Ciocalteu phenol reagent. The extracts of non-supplemented feed and both beta-glucan fractions (obtained with acidified methanol) revealed very low reactivity vs. Folin-Ciocalteu reagent at range of 0.2–0.4 mg of gallic acid equivalent per gram of dry basis (GAE/ g d.b.). The feed samples with beta-glucan fraction of high molar mass (FβGh+) showed significantly higher polyphenols content (more than twice) than feed supplemented with low molar mass (FβGl+) (0.2 and 0.48 mg GAE/1 g d.b. respectively). Similar low values were obtained by Inglett et al. 2012 [22] for 95% pure oat beta-glucan from Megazyme International Ltd. Authors recorded value of 0.84 GAE mg/g sample and the higher value could be obtained due to different extraction method which was used for standard samples obtained by Megazyme and prolonged time of incubation (2 h). Therefore, the overall activity vs. Folin-Ciocalteu reagent (total polyphenols content) can be neglected in all samples.

The antioxidant capacity (AC) measured as radical scavenging activity vs. 2,2-diphenyl-1-picrylhydrazyl free radical showed that the highest AC was observed for control feed at the level of 7.33 μmol of Trolox equivalent. Both beta-glucan samples and feed with low molar mass beta-glucan additive were not significantly different of 5.0 μmol of Trolox equivalent for 1g of d.b. Additionally, the feeds supplemented with beta-glucans of different molar mass were not significantly different in regard to their antioxidant capacity.

The results confirmed that purified beta-glucan fractions do not possess any pronounced polyphenols content or antioxidant activity vs DPPH. Therefore, any improvement in antioxidant status of tissues after ingestion of feed supplemented with beta-glucans is triggered by mechanisms not involving the activity of polyphenols.

### 3.2. Feed Intake, Body Weight Gains and the Activity of Selected Liver Enzymes

**Figure 2:**
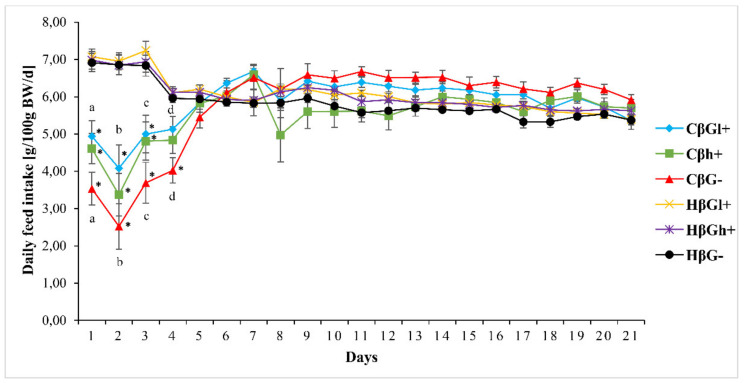
Mean daily feed intake in rats during the experiment. * significantly different between the *colitis* (C) and healthy control groups (H) at the same time point and the same feed according to the Tukey *post-hoc* test (*p* < 0.01); ^a–d^ Significant difference between groups (CβGl+ vs CβG−) according to the Tukey *post-hoc* test (*p* < 0.01). The same letters show statistically significant results at the same time point. (a—*p* < 0.01, b—*p* < 0.004, c—*p* < 0.01, d—*p* < 0.001).

Average feed intake in rats from control groups did not differ significantly during the whole experiment (at the beginning 6.99 ± 0.13 g/d/100 g b.w., at the end 5.46 ± 0.07 g/d/100 g b.w.), whereas rats with TNBS-induced *colitis* inflammatory bowel disease, consumed significantly less feed on the next day after the treatment (4.35 ± 0.24 g/d/100 g b.w.), regardless of the type of feed consumed. On the third day after treatment, the daily intake of feed in all groups with *colitis* increased and on the fifth day after the administration of TNBS it was already the same as in the control groups (5.89 ± 0.08 g/d/100 g b.w.). It should be noted that during the first 5 days after TNBS administration the feed intake in rats in the CβGl+ group was significantly higher than in the CβG− group (Figure 2). 

The initial body weight of all 150 animals was uniform and amounted to 414.0 ± 1.29 g. The percentage weight gains of rats from control groups (H), regardless of nutritional intervention, did not differ significantly and their dynamics indicated good health of animals and proper assimilation of feed components. The weight gains in rats with experimentally induced *colitis* (C) after 7 and 21 days were significantly lower and in animals from CβGl+ group, the difference was the lowest after 21 days of feeding (Table 1).

The activity of alanine aminotransferase (ALT), aspartate aminotransferase (AST) and alkaline phosphatase (ALP) in blood plasma did not show significant differences among particular experimental groups (Table 2), which indicates low hepatotoxicity.

### 3.3. Hematological Parameters and Cytometric Analysis

The values of hematological parameters are presented in Table 3. As the analysis of variance showed, the number of white blood cells (WBC) and their individual types (lymphocytes (LYM), monocytes and eosinophils (MID) and granulocytes (GRA)) depended significantly on the time elapsed since the administration of TNBS to animals (ANOVA, *p* < 0.001, *p* < 0.01, *p* < 0.001, *p* < 0.001, respectively), with the lowest values in rats after 3 days of experiment. The analysis of variance also showed a significant influence of the type of feed fed to animals on the WBC and LYM values, the lowest value of which was found in animals fed with βGh+ feed (ANOVA, *p* < 0.05 for both parameters). The highest MID value was found in rats with *colitis* after 7 days of experiment in the group fed diet supplemented with low molar mass beta-glucan (CβGl+), which was confirmed by a highly significant interaction between the duration of dietary supplementation and its type and the occurrence of inflammation (ANOVA, *p* < 0.01). Experimental factors had a slight effect on hematocrit (HCT), the value of which depended only on the type of feed (ANOVA; *p* < 0.05), however, *post-hoc* Tukey analysis did not show statistically significant differences between experimental groups in HCT values. The analysis of variance showed that the number of erythrocytes (RBC), as well as the values of red cell parameters, including mean corpuscular volume (MCV), mean corpuscular hemoglobin (MCH) and red cell distribution width (RDW), significantly depended on the duration of the experiment after TNBS administration (ANOVA, *p* < 0.001 for all). The RBC value after 7 and 21 days of feeding was higher in rats in HβGl+ group compared to the value recorded after 3 days (*post-hoc* Tukey test, *p* < 0.05). The analysis of variance showed that MCV and mean corpuscular hemoglobin concentration (MCHC) were not significantly affected by *colitis* but these parameters significantly depended on the diet (ANOVA, *p* < 0.001, *p* < 0.001, respectively). The highest MCV values were found in animals that consumed feed without beta-glucans, while the lowest values were found in animals fed on βGl+ diet, whereas in the case of MCHC the opposite relationship was observed. It should be noted that the lowest value of MCHC was found in rats in the CβG- group after 3 days of experiment.

The degree of red cell distribution width (RDWc) was influenced by all experimental factors—length of the feeding period and induced inflammation as well as the type of feed consumed. As shown by ANOVA, the highest increase in the RDWc value in blood was noted in animals 21 days after TNBS administration (ANOVA, *p* < 0.05). Higher values of RDWc were characteristic for animals from *colitis* groups consuming βGl+ supplemented feed (CβGl+) (ANOVA; *p* < 0.01, *p* < 0.001, respectively).

**Figure 3:**
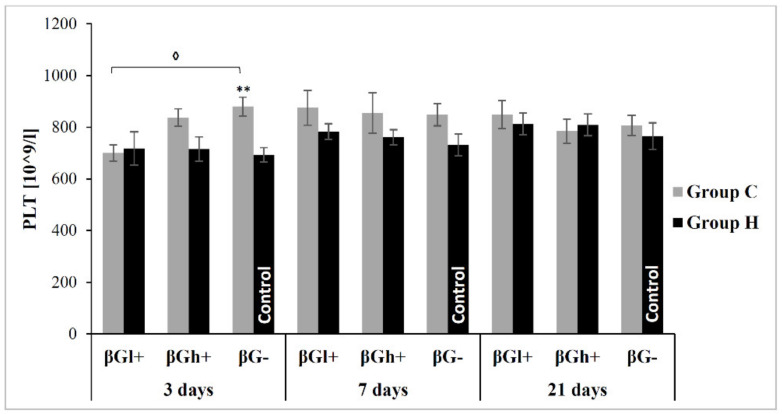
Platelet count (PLT). * Significantly different from control group (HβG−) at the same time point according to the Dunnett *post-hoc* test (** *p* < 0.01) **^◊^** Significantly different from *colitis* group at the same time point according to the Tukey *post-hoc* test (**^◊^** *p* < 0.05).

As the results of variance analysis indicate, platelet count (PLT) was significantly higher in rats from *colitis* groups (ANOVA, *p* < 0.01) (Figure 3). Three days after administration of TNBS, PLT was the highest in CβG− group, significantly higher compared to both the control (HβG−) and CβGl+ groups (Tukey *post-hoc* test, *p* < 0.01 and *p* < 0.05, respectively). After 7 and 21 days of feeding, the PLT value did not differ among particular groups.

**Figure 4:**
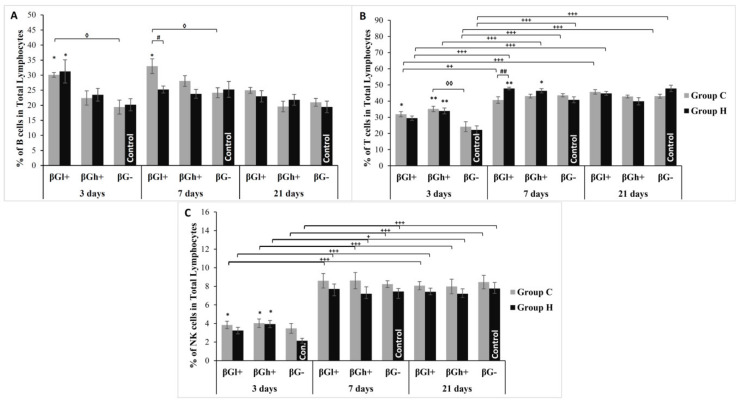
Changes in the percentage of peripheral blood lymphocyte subpopulations (mean ± SE). (**A**)—percent of B cells in total lymphocytes; (**B**)—percent of T cells in total lymphocytes, (**C**)—percent of natural killer (NK) cells in total lymphocytes. * Significantly different from control group (HβG−) at the same time point according to the Dunnett *post-hoc* test (* *p* < 0.05, ** *p* < 0.01). # Significantly different between the *colitis* and control groups at the same time point and the same feed according to the Tukey *post-hoc* test (# *p* < 0.05, ## *p* < 0.01, ### *p* < 0.001). **^◊^** Significantly different from *colitis* group at the same time point according to the Tukey *post-hoc* test (**^◊^** *p* < 0.05, **^◊◊^** *p* < 0.01). + Significantly different from the same subgroups at another time point according to the Tukey *post-hoc* test (+ *p* < 0.05, ++ *p* < 0.01, +++ *p* < 0.001).

The percentage share of specific subpopulations of lymphocytes in the population of PBMC is presented in Figure 4. The percentage of T cells in the lymphocyte population increased at individual time points of the experiment. As the results of the analysis of variance indicate, the percentage share of T cells was also dependent on the type of feed (ANOVA, *p* < 0.001), assuming higher values in both groups that consumed feed with beta-glucans. This was particularly evident after 3 days of supplementation, when significantly more of these cells were recorded in CβGl+ and CβGh+ groups. A similar relationship was also observed for the B cell population. In this case, the analysis of variance showed a significant effect of time elapsed since the administration of TNBS as well as the type of supplementation used (ANOVA, *p* < 0.001). The highest percentage of B lymphocytes in the total pool of lymphocytes was recorded in βGl+ group after 3 days of the experiment.

The analysis of variance showed that the duration of the experiment (ANOVA, *p* < 0.001) and colon inflammation (ANOVA, *p* < 0.01) also had a significant impact on the percentage of NK cells in the lymphocyte population. Higher percentages of NK cells were found in animals after 7 and 21 days of the experiment. After 3 days from the administration of TNBS, a higher percentage of NK cells was observed in groups of animals with *colitis* CβGl+ and CβGh+ vs. HβG− control (Tukey *post-hoc* test, *p* < 0.05 for both). The stimulating effect of beta-glucans on NK cells is confirmed by an increased percentage of these cells also in groups of animals without *colitis* HβGh+ (Tukey *post-hoc* test, *p* < 0.05).

### 3.4. Parameters of Redox Balance in Peripheral Blood

**Figure 5:**
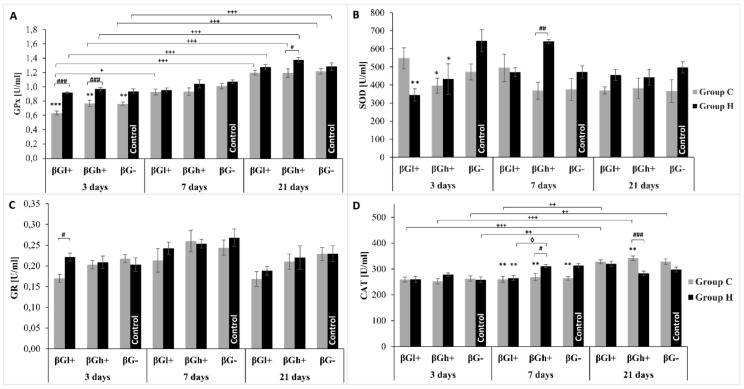
Activity of antioxidant enzymes detected in whole blood. The data are presented as mean ± SE. (**A**)—glutathione peroxidase (GPx) activity, (**B**)—superoxide dismutase (SOD) activity, (**C**)—glutathione reductase (GR) activity, (**D**)—catalase activity (CAT). * Significantly different from control group (HβG−) at the same time point according to the Dunnett *post-hoc* test (* *p* < 0.05, ** *p* < 0.01, *** *p* < 0.001). # Significantly different between the *colitis* and control groups at the same time point and the same feed according to the Tukey *post-hoc* test (# *p* < 0.05, ## *p* < 0.01, ### *p* < 0.001). + Significantly different from the same subgroups at another time point according to the Tukey *post-hoc* test (+ *p* < 0.05, ++ *p* < 0.01, +++ *p* < 0.001).

The activity of key antioxidant enzymes involved in the maintenance of redox balance is presented in Figure 5. As the analysis of variance showed, the activity of SOD detected in blood cells lysate was lower in rats with TNBS-induced *colitis* (ANOVA, *p* < 0.01), whereas feeding intervention with added oat beta-glucans resulted in a change in the activity of this enzyme after 3 and 7 days of feeding, which was confirmed by ANOVA analysis. After 3 days of feeding the experimental diet, the activity of SOD in HβGl+ group rats was significantly lower compared to HβG- control group (ANOVA, *p* < 0.01), whereas after 7 days of feeding intervention in HβGh+ group animals the activity of this enzyme in relation to the value found in CβGh+ group increased significantly (ANOVA, *p* < 0.01). After 21 days of feeding, the activity of SOD did not differ between particular groups.

The analysis of variance showed that the inflammation induced by TNBS in the large intestine did not have a significant effect on the catalase activity detected in blood after 3 days of feeding, whereas after 7 days a decrease in the activity of this enzyme was observed in the CβGh+ and CβG- groups (ANOVA, *p* < 0.05 and *p* < 0.01, respectively) and after 21 days an increase in the CβGh+ group was noted in relation to the respective control groups. It should be noted that the highest CAT activity was found in blood of rats with *colitis* after 21 days of experiment, which was confirmed by a highly significant interaction between these two factors affecting CAT (ANOVA, *p* < 0.001).

The glutathione reductase activity also did not differ significantly between *colitis* and control rats fed for 7 or 21 days with βGl+ and βGh+ or βG− feed, whereas after 3 days of dietary intervention the activity of this enzyme was found to be significantly lower in CβGl+ group animals compared to HβGl+ group. The duration of nutritional intervention (ANOVA, *p* < 0.001) was a significant factor. The analysis of variance also showed a significant influence of diet supplementation with low molar mass oat beta-glucan on the decrease of GR activity (ANOVA, *p* < 0.01).

As the results of variance analysis indicate, glutathione peroxidase activity was significantly lower in rats from *colitis* groups (ANOVA, *p* < 0.001) and higher after 21 days of nutritional intervention (ANOVA, *p* < 0.001), which was reflected in significant interaction between the two factors (ANOVA, *p* < 0.05). The effect of feed used with or without beta-glucans was less significant (ANOVA, *p* < 0.01), however, it manifested itself significantly after 3 days of nutritional intervention. The greatest differences in GPx activity between *colitis* and control animals were found using feed with or without beta-glucans regardless of their molar mass (ANOVA, *p* < 0.001).

**Figure 6:**
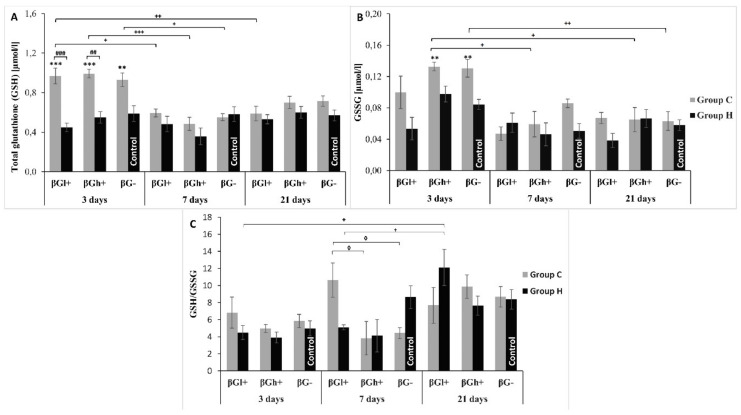
Glutathione metabolism in whole blood. The data are presented as mean ± SE. (**A**)—total glutathione concentration (GSH), (**B**)—glutathione disulfide concentration, (**C**)—total glutathione to glutathione disulfide concentration ratio. * Significantly different from control group (HβG−) at the same time point according to the Dunnett *post-hoc* test (** *p* < 0.01, *** *p* < 0.001). # Significantly different between the *colitis* and control groups at the same time point and the same feed according to the Tukey *post-hoc* test ## *p* < 0.01, ### *p* < 0.001). + Significantly different from the same subgroups at another time point according to the Tukey *post-hoc* test (+ *p* < 0.05, ++ *p* < 0.01, +++ *p* < 0.001). **^◊^** Significantly different from *colitis* group at the same time point according to the Tukey *post-hoc* test (**^◊^** *p* < 0.05).

The blood concentration of total glutathione (GSH), glutathione disulfate (GSSG) and the ratio of total glutathione (GSH) to oxidized one (GSH/GSSG) are also the markers of the redox processes (Figure 6). As the three-factor analysis of variance showed, both colon inflammation and length of dietary intervention period had a significant effect on GSH (ANOVA *p* < 0.001 for both factors) with higher values in rats with *colitis* and after 3 days of experimental feeding. The interaction between the two factors was also significant (ANOVA, *p* < 0.001). Induced *colitis* caused a significant increase in GSH concentration after 3 days in all feeding subgroups (for CβGh+ and CβGl+ groups *p* < 0.001; for CβG− *post-hoc* Tukey test, *p* < 0.01).

GSSG concentration depended significantly on the duration of nutritional intervention (ANOVA, *p* < 0.001) and was the highest after 3 days. Furthermore, the inflammatory state also played a significant role in GSSG concentration. Higher level of GSSG was observed in rats in TNBS-induced *colitis* than in healthy controls on day 3 of the experiment (ANOVA, *p* < 0.001). Reduced GSSG concentration was found in animals fed with βGl+ feed (ANOVA, *p* < 0.05).

The ratio of blood total glutathione to oxidized glutathione depended significantly on the duration of the experiment (ANOVA, *p* < 0.001) with the highest value after 21 days. This parameter depended also on the type of feed the animals were fed (ANOVA, *p* < 0.05) with the highest value when using βGl+ feed. The analysis of variance also showed significant interaction between all three examined factors (ANOVA, *p* < 0.01). A significantly higher value of this parameter was also found after 7 days in CβGl+ group as compared to CβGh+ and CβG− (*post-hoc* Tukey test, *p* < 0.05, for both).

**Figure 7:**
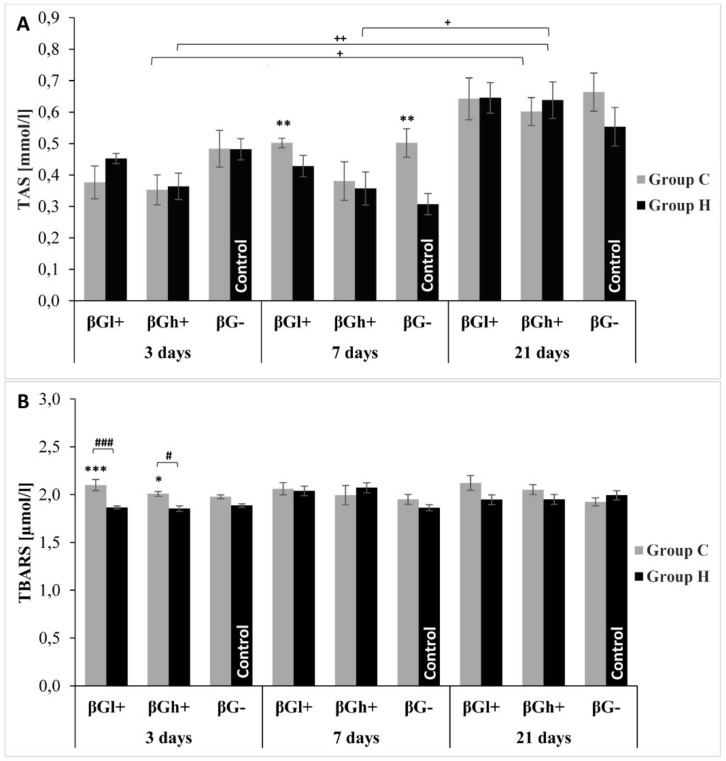
(**A**)—total antioxidant status in plasma (TAS); (**B**)—the level of thiobarbituric acid reactive substances (TBARS) in plasma. The data are presented as mean ± SE. * Significantly different from control group (HβG−) at the same time point according to the Dunnett *post-hoc* test (* *p* < 0.05, ** *p* < 0.01, *** *p* < 0.001). # Significantly different between the *colitis* and control groups at the same time point and the same feed according to the Tukey *post-hoc* test (# *p* < 0.05, ### *p* < 0.001). + Significantly different from the same subgroups at another time point according to the Tukey *post-hoc* test (+ *p* < 0.05, ++ *p* < 0.01).

The total antioxidant potential of blood plasma (Figure 7) is an indicator of oxidative stress regulated by various mechanisms. TAS level, in this experiment, was only dependent on the duration of the experiment (ANOVA, *p* < 0.001) with the highest concentration detected after 21 days. The value of this parameter did not differ significantly between rats with *colitis* and control ones independently of the feed consumed, except for animals fed for 7 days. In this case, rats with *colitis* fed without beta-glucans had a significantly higher TAS value (Tukey *post-hoc* test, *p* < 0.01).

In the present study the concentration of thiobarbituric acid reactive substances (TBARS) was used to investigate the level of lipid peroxidation in blood plasma. Inflammation of the large intestine caused an increase in TBARS concentration (ANOVA, *p* < 0.001). The value of this parameter also depended on the type of dietary intervention applied (ANOVA, *p* < 0.01) and was higher in rats fed with βGl+ feed. No differences due to *colitis* or the type of feed consumed were found after 7 and 21 days of the experiment, whereas after 3 days of feeding, the TBARS concentration in rats with *colitis* was significantly higher in relation to animals from control groups when using feed with βGl+ (*p* < 0.001) and βGh+ (Tukey *post-hoc* test, *p* < 0.05).

### 3.5. Fisher’s Linear Discriminant Analysis (FLD)

**Figure 8:**
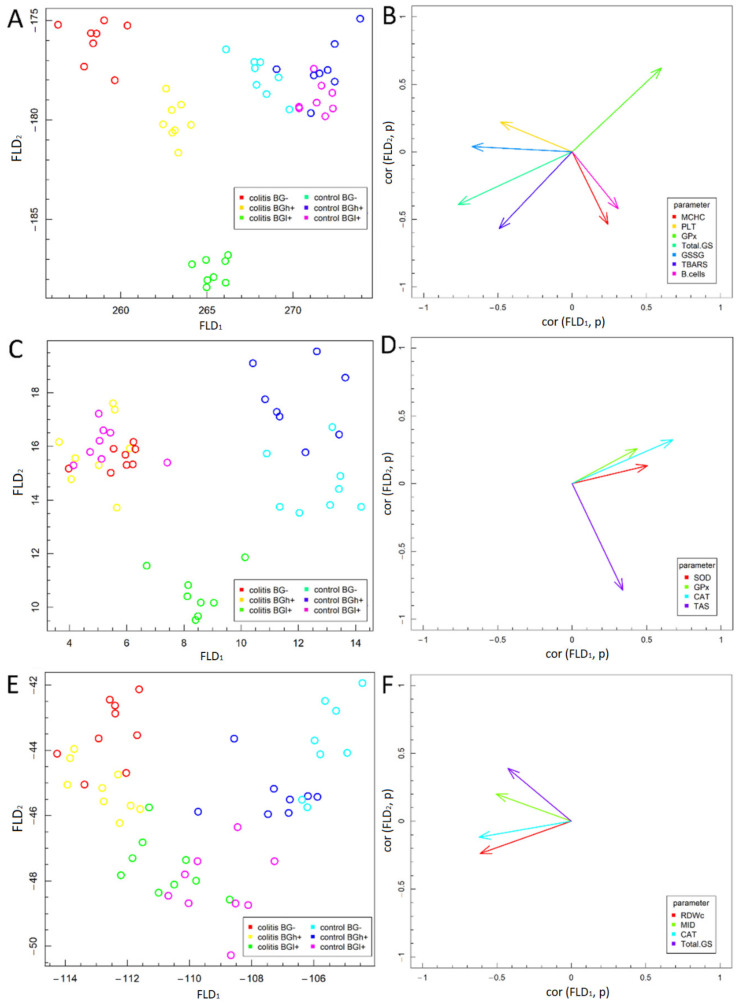
Fisher’s Linear Discriminant (FLD) Analysis: (**A**,**C**,**E**) experimental data on the plane spanned by two the most data separating FLDs and (**B**,**D**,**F**) parameters contributing the most to FLDs. (**A**,**B**)—3 days; (**C**,**D**)—7 days; (**E**,**F**)—21 days.

The FLD analysis of the experimental factors examined is presented in Figure 8. This analysis was used in order to find linear combinations of analyzed biochemical and hematological parameters that allow for the best separation of the groups of animals at three time points selected in the experimental model. FLD method is useful for selection of parameters which differentiate the data corresponding to different experimental groups [23]. Based on data division to different groups, FLD method provides linear combination of parameters used in the analysis, such that separation of between data groups is the best possible among all linear combinations of parameters. Data groups at three time points considered in the study are shown in Figure 8A,C,E. In each of the figures, 6 experimental groups have been distinguished—data are presented in the space spanned by linear combinations of parameters (Fisher Linear Discriminants—FLDs), that is, FLD_1_, FLD_2_ which are the best separating those predefined groups.

The vectors visible in Figure 8B,D,F show the direction in which relevant parameters determine the separation of experimental groups at three time points—3 days (A and B), 7 days (C and D) and 21 days (E and F). High values of a parameter corresponding to a particular vector, tends the data to be shifted along direction determined by the vector. The conducted analyses complement the ANOVA analysis and allow to summarize the obtained results.

The FLD analysis of animal parameters performed for data collected 3 days after *colitis* induction (Figure 8A) showed that the factors most differentiating these experimental groups were—MCHC, PLT, GPx, GSH, GSSG, TBARS and percentage of B lymphocytes. FLD results showed that it was possible to determine a combination of the above parameters, which allow to separate control groups (HβG−, HβGl+ and HβGh+) from *colitis* groups (CβG−, CβGl+, CβGh+) in a horizontal plane (FLD_1_). The vertical plane (FLD_2_) allowed to separate the *colitis* groups from each other (Figure 8A.). GSSG, TBARS and PLT had the greatest influence on FLD_1_, whereas MCHC had a significant influence on FLD_2_, while GSH, GPx and B lymphocytes are important for both FLDs (Figure 8B). On the basis of LDA it can be concluded that the βGl+ *colitis* group was characterized by significantly higher MCHC and TBARS levels, percentage of B lymphocytes and lower PLT count and GSSG concentration in comparison to the *colitis* group fed without beta-glucans. Higher GSH, GSSG and TBARS concentrations and lower GPx activity were also characteristic for *colitis* groups.

After 7 days from the induction of *colitis* (Figure 8C), a clear separation of the inflammation groups from all control groups (that was observed after 3 days) was already much more difficult, which proves the effective regeneration process in the large intestine, manifested by a much smaller systemic reaction. CAT, SOD and GPx activity had the greatest influence on FLD_1_, whereas TAS parameter influenced FLD_2_ values (Figure 8D.). FLD shows that CβGl+ group remains clearly separated from others. It is separated from the control groups by FLD_1_ and from the other *colitis* groups (CβG− and CβGh+) by FLD_2_. Also HβG− and HβGh+ groups are quite well separated from the other groups by FLD_1_. On the other hand, HβGl+ group is very similar in profile to CβG− and CβGh+ groups, which proves the ability of low molar mass oat beta-glucan to inhibit CAT, SOD and GPx activity in healthy animals, with concurrent high TAS level (Figure 8C). The highest SOD activity is characteristic for the control group with high molar mass beta-glucan (HβGh+).

After 21 days from the administration of TNBS (Figure 8E), the parameters examined in our experiment do not differentiate between individual experimental groups anymore, which proves very small systemic effects of *colitis*. Few of the examined parameters cause separation of CβG− and CβGh+ groups from control groups by FLD_1_. The CβGl+ group is no longer separated from the control groups by FLD_1_ but is still separated from the other *colitis* groups by FLD_2_, which proves the greatest effectiveness in removing systemic effects of colon inflammation by low molar mass beta-glucan (Figure 8E). After 21 days of nutritional intervention, only four parameters differentiating the experimental groups were left and to a much lesser extent than at the earlier time points (3 and 7 days). CAT activity had the greatest influence on FLD_1_, whereas RDWc, MID and GSH parameters affected both FLDs (Figure 8F.). Based on FLD analysis, we showed that MID, GSH and CAT values characteristic for CβG− and CβGh+ groups are slightly higher compared to control groups and CβGl+, whereas RDWc in HβG− group is significantly lower compared to *colitis* groups.

## 4. Discussion

The aim of the present study was to verify whether the consumption of feed with 1% addition of oat beta-glucans of different molar mass by rats with experimentally induced colon inflammation restores the redox balance in the organism. To investigate the antioxidant potential we measured the concentration and activity of chosen markers in peripheral blood of animals subjected to different experimental groups. Furthermore, our study was aimed at determining whether the effect of supplementation with oat beta-glucans, if any, depends on the duration of the dietary intervention and/or the molar mass of these polysaccharides. We decided to use chemically pure oat beta-glucan factions in order to eliminate the potential effect of other bioactive compounds, that could influence the results obtained. The whole oat cereals consist of an abundance of different bioactive compounds, typical for cereal grains, that present high biological activity. High unsaturated fatty acids, tocopherols, carotenoids, polyphenols of specific groups called avenantramides, steroidal saponins as well as specific proteins and polysaccharides like beta-glucan provide complex interaction with metabolism and trigger different responses due to their specific chemical structure and hence, receptors bonding [24,25,26] in the extensive review provided the substantial discussion proving that the consumption of the whole oat is beneficial not only due to high beta-glucan content but also or even predominantly, due to the abundance of other compounds with strong antioxidative properties. He et al. [27] analyzed the impact of the intake of oats vs. purified beta-glucan using meta-analysis of randomized controlled trials evaluating HbA1c levels, fasting glucose and insulin sensitivity. The authors confirmed the previous observation that increased consumption of whole oats and oat bran but not their extracts, are associated with lower HbA1c, fasting glucose and fasting insulin in type 2 diabetes (T2D), hyperlipidaemic and overweight subjects and this effect was pronounced especially in people with T2D. Therefore, to properly evaluate the impact of purified beta-glucan fractions and their interactions with metabolic pathways it is necessary to minimize the important impact of other compounds, mainly those showing antioxidant effect.

The literature data and our study showed that in terms of morphology and histopathology, the TNBS model of colon inflammation used in the present study is adequate for early stage of Crohn’s disease (CD) in humans [18,28], data not published]. The rectal administration of trinitrobenzene sulfonic acid (TNBS) results in a strictly localized trans-wall inflammation causing local immune response with dense lymphocyte infiltration and secretion of proinflammatory cytokines in the whole wall of the large intestine [29,30]. The occurrence of typical for CD features in the large intestine of rats included in our experiment was confirmed by histological analysis of the pathologically altered sections of the large intestine (rectum) and the analysis of the concentration of proinflammatory cytokines performed on the same material. The results of these analyses are described in the other articles of our team [18], data not published]. It should be noted that TNBS-induced inflammation is a model of an early, yet minimally invasive stage of CD, as evidenced by the lack of increase in plasma hepatic aminotransferases observed in our study in animals with *colitis*. Moreover, the early stage of colon inflammation in rats included in this study was evidenced by good general health, despite quite significant macroscopic changes in the rectum; food intake comparable to control animals and normal weight gains already observed on the fifth day after the inflammation was induced. Results of other studies on IBD demonstrated that inflammation of the intestines is often associated with generalized parenteral symptoms of other internal organs, including liver. Elevated plasma aminotransferase levels found in patients with advanced stage of IBD result from damage to this organ, mainly fibrosis [31,32].

Our previous study showed macro- and microscopic changes in the colon tissue that confirm acute inflammation developed after administration of TNBS. The full-walled inflammation lesions were located only in the final segment of the colon, that is, in the rectum [18]. It should be emphasized that the rectum in rats is a section of the intestine where the immune system is highly developed, thus the focus of inflammation is more quickly silenced and the systemic inflammation develops more slowly. Changes in the profile of lymphocyte subpopulation in blood of all animals, including those from control groups, indicate a significant influence of rectal surgery itself, as 3 days after the administration of TNBS, rats from all groups showed a significantly lower percentage of NK cells and T lymphocytes in relation to later study periods. The above results confirm that colon inflammation induced through rectal administration of ethanol picrylsulfonic acid solution (TNBS) causes only local inflammation of the rectum with short-term parenteral clinical symptoms and no systemic inflammation. Nevertheless, in peripheral blood there are changes in both morphotic elements and redox balance markers.

In inflammatory bowel diseases, especially CD, a sensitive marker measured in peripheral blood is the platelet count (PLT). Platelets play a significant role in the pathogenesis of all inflammatory conditions and in IBD they significantly affect the intestinal mucosa by secreted mediators [33], whereas the concentration of compounds originating from PLT increases with the intensity of CD, which is an important predictor of both the disease severity and effectiveness of the therapy used [34]. This is confirmed by the results of many human studies, which indicate an increase in platelet count during the active phase of ulcerative colitis or CD [35,36,37]. Also model studies in animals with induced colon inflammation prove this relation [38]. An increase in platelet count and activity in the course of IBD is likely to result from exacerbation of the inflammatory process accompanied by oxidative stress related to increased number of reactive oxygen species. Moreover, platelets are the main factor contributing to the thrombotic process in the active phase of IBD, during which bleeding often occurs [39]. The results of our study indicate that the increase in the number of PLT and their activation correlates with an increase in lipid peroxidation, which resulted in the observed increased concentration of plasma TBARS, the highest after 3 days of TNBS administration. This hypothesis is confirmed by a positive correlation between PLT and TBARS at this time point in rats with *colitis* fed control feed without beta-glucan (*p* < 0.004). Dietary supplementation with high molar mass beta-glucan significantly reduced these correlations and after 7 and 21 days the significant correlations between PLT and plasma TBARS in all animal groups were not found, which confirms the hypothesis of significant participation of these blood morphotic elements only in the initial phase of CD and effective elimination of these lesions by oat beta-glucan [18]. Mechanism responsible for the observed increase in lipid peroxidation is related to myeloperoxidase (MPO), which is a lysosomal enzyme present in the cells of the immune system. Under inflammatory conditions in the gastrointestinal tract, a well-described phenomenon is an increase in its activity, which results in the induction of inflammatory reactions and acceleration of ROS synthesis and secretion. Available literature data demonstrate that IBD is associated with an imbalance between increased ROS and decreased antioxidant activity, which may explain, at least in part, many of the clinical pathophysiological features in IBD (both CD and UC) patients [40]. Additionally, the inflammatory process induces oxidative stress and reduces cellular antioxidant capacity [41]. In our study we observed higher TBARS in CβGl+ rats after 3 days of TNBS treatment. It seems that the strong oxidative stress generated in the acute phase of inflammation, at the beginning of the TNBS treatment (3 days after TNBS administration to rats) was so significant that it exceeded the protective effect of beta-glucans in the plasma, regardless of their chemical structure. Therefore, the level of plasma TBARS at this time point is so high in comparison with the physiological conditions. In reaction to oxidative stress generated by TNBS treatment, rats tissues responded by producing antioxidants but oxidative stress depleted tissue antioxidant resources and dismissed the ability to produce more exogenic antioxidants, therefore leading to lower antioxidant levels, observed also in blood. We suggest that the longer supplementation with beta-glucans is needed to reduce lipid peroxidation by protecting cell integrity in the damaged colon mucosa. Previous results of our team demonstrated a significant increase in the colon activity of MPO in animals with *colitis* caused by LPS or TNBS [17,18]. It should be added that MPO is a common indicator of free radical damage in patients with IBD. The increased oxidative stress in the organism 3 days after the administration of TNBS is also confirmed by the significantly reduced GSH/GSSG ratio detected in blood cell lysates.

The pathophysiology of IBD is closely related to oxidative stress caused mainly by overproduction of reactive oxygen and nitrogen species (ROS and RNS) [40]. Excessive ROS production induces and amplifies the cascade of signals recruiting numerous inflammatory markers, including interleukins and adhesion proteins [42]. Generation of excess free radicals due to endogenous (e.g., changes in mitochondrial metabolism) and exogenous factors (e.g., food xenobiotics or drugs) leading to the activation of antioxidant defense mechanisms in the gastrointestinal tract, is at least partially responsible for the initiation and progression of IBD. The antioxidant defense mechanisms, which include intracellular enzymes (SOD, GPX, CAT) and non-enzymatic antioxidants like glutathione, restore the redox balance under physiological conditions. If the antioxidant defense mechanisms are impaired, inflammation develops and results in IBD progression [28].

For this reason, natural ingredients, such as beta-glucans, with not only anti-inflammatory but above all antioxidant effects are sought to support the therapy of these gastrointestinal disorders or to alleviate the course of inflammation associated with IBD. Our previous studies have demonstrated that oat beta-glucan show immunomodulating, anti-inflammatory and antioxidant properties [16,17,18,43]. In our study, the diet does not contain any sources of polyphenols and simultaneously the beta-glucan preparations themselves as highly purified are not contaminated with polyphenols that were showed evidence in the TPC method. However, the DPPH assay performed in this study to analyze the direct antioxidant properties of beta-glucan preparations of various molar masses isolated from oat grains and added to the feed for rats did not confirm such properties. In addition, we did not demonstrate the presence of polyphenols with antioxidant properties in these preparations. The studies by other authors showed that beta-glucan from barley grains is characterized by higher free radical scavenging properties, whereas beta-glucans from oats or yeasts do not have such properties [44]. Recent studies, however, demonstrated that the ability to scavenge free radicals by barley beta-glucan is also negligible [45]. The results of our in vivo studies indicate that despite the lack of direct antioxidant action of oat beta-glucans, they have an indirect antioxidant effect on the body, especially during ongoing colon inflammation. Therefore, it can be argued that these oat polysaccharides, regardless of their molar mass, are inert compounds in terms of their ability to scavenge free radicals and their antioxidant action in the body is caused by a metabolic response which restores the redox balance. The indirect action of these polysaccharides consists in their binding to specific membrane receptors of immune cells, especially antigen-presenting cells (macrophages, dendritic cells and B lymphocytes) and these mechanisms, as shown by our research presented in this article, depend on the length of time that passed since the administration of TNBS and the molar mass of beta-glucan.

The activation of immune cells, especially macrophages and neutrophils, can cause the production of superoxide and nitric oxide by NADPH oxidase (NOX) and nitric oxide synthase (NOS) as well as the synthesis of peroxynitrite with oxidizing properties. It was also demonstrated that both enzymes are involved in the pathogenesis of IBD, particularly in the initiation and progression of Crohn’s disease [28]. Moreover, activation of macrophages by beta-glucans results in the release of chemical mediators, including ROS, nitric oxide, hydrolytic enzymes and proinflammatory cytokines, such as TNF-α [46]. Binding of beta-glucans to specific receptors also enables phagocytosis of their molecules by macrophages; the accompanying changes in macrophage functions (e.g., increased secretion of pro-inflammatory cytokines) are the mechanism through which beta-glucans affect the inflammation, including its initiation [47]. This was most probably the case in our in vivo experiment, when 3 days after the administration of TNBS, the redox balance parameters in peripheral blood significantly changed. In rats with induced *colitis*, apart from the already mentioned increase in platelet count and increase in lipid peroxidation (TBARS), the concentration of total and oxidized glutathione increased and the activity of glutathione peroxidase decreased. In parallel, animals in the *colitis* group showed increased concentrations of proinflammatory cytokines in the colon tissue, which has been described in our previous article [18]. The ratio of reduced to oxidized glutathione is an indicator of oxidative stress status of a cell and an excellent measure of potential therapeutic effectiveness in maintaining the redox balance. Under physiological conditions, the reduced form of GSH is up to 98% of the total glutathione present in the cell. The reduction of GSH/GSSG ratio is characteristic for many diseases, including IBD [28]. In our study, we calculated the ratio of total glutathione to oxidized glutathione, however, such a ratio also reflects the oxidative status of cells. The lowest ratio of GSH/GSSG was found 3 days after administration of TNBS but this value was significantly increased in rats fed with low molar mass beta-glucan.

The pathogenesis of IBD involves complex mechanisms acting through different pathways. One of them is imbalance between auxiliary T lymphocytes (Th) and regulatory lymphocytes (Treg). CD is characterized by excessive production of proinflammatory cytokines such as IL-12, IL-17, IL-23, interferon-gamma and TNF-alpha by Th1 lymphocytes, which leads to damage of the intestinal mucosa barrier [28]. Beta-glucans bind with TLR receptors present on dendritic cells causing the decrease in the synthesis of these cytokines [14], also the administration of these polysaccharides to obese mice with diabetes decreased the concentration of TNF-alpha, IL-6, IL-1β and increased the level of anti-inflammatory factors [48].

The already published results of our team indicate a very large increase in the concentration of pro-inflammatory cytokines (IL-1, IL-6, IL-12) and a decrease in the concentration of anti-inflammatory cytokine (IL-10) in the inflammatory lesion of the colon wall, whereas the supply of oat beta-glucans contained in the feed significantly decreased the concentration of pro-inflammatory cytokines 21 days after TNBS administration [18]. It should also be emphasized that the anti-inflammatory effects of low molar mass beta-glucan were significantly stronger. This was probably due to the fact that as a result of local inflammation, the mucous membrane of the large intestine was damaged, which resulted in unsealing of connections between the cells, which enabled low-molar beta-glucan to penetrate the plate of the proper wall of the large intestine and further into blood. The possibility of passing from the intestinal lumen to the blood of small size soluble beta-glucans was confirmed by other authors [12]. There is also likely to be another mechanism whereby the small molecules of these polysaccharides bind more easily to the membrane receptors of the cells compared to the large molecules, which must first be broken down by the feeding cells into smaller ones and can only connect to the corresponding receptors. Our previous results indicate an inflammation reduction in the intestine in animals fed with oat beta-glucan; however the results of parameters measured in the peripheral blood and presented in this article do not allow us to conclude about the systemic immunomodulatory effect of beta-glucans, which are administered with diet.

Nevertheless, given the pathomechanism of IBD and the results of our previous studies [16,17,18] one can state that the immunomodulatory activity of beta-glucans is able to alleviate the local inflammation observed in the intestines and inhibit the activity of locally distributed immune cells, including macrophages and neutrophils, which are crucial in inducing oxidative stress, thus reducing the production of free radicals and oxidative damage to the intestinal mucosa barrier.

## 5. Conclusions

Plant isolates usually possess residual antioxidant capacity or extant polyphenols mainly due to extraction method used to recover this particular compound from plant matrix. Some previous results obtained by other Authors, which indicated antioxidative effect of consumed cereal beta-glucans fraction, could be biased as fractions used were not pure. Therefore, after isolation of beta-glucan from oat bran matrix it was important to verify if there is any significant residual antioxidant capacity left. Exclusion of polyphenols content and radicals scavenging activity of used oat beta-glucan fractions allowed to conclude upon the obtained results about indirect antioxidative action of consumed oat beta-glucans.

Chemically pure preparations of oat beta-glucan do not have the ability to scavenge free radicals, while in the body of model animals with colon inflammation, consuming feed with their addition, they show antioxidant effects. This suggests that the mechanism of action of these cereal polysaccharides is not direct but consists in agonistic binding of immune cells to membrane receptors, which results in an increased antioxidant response. This response occurs after a short period of time after colon inflammation is induced and decreases with time, which proves the effective therapeutic effect of these compounds. Our results also showed that oat beta-glucan of low molar mass is more effective in removing systemic effects of *colitis*, which is also associated, as our other studies show, with its stronger effect observed in the very tissue of the colon.

## Figures and Tables

**Table 1:** Percentage changes in the body weight (mean ± SE).

Time [Days]	Healthy Group			*Colitis* Group		
	HβG−	HβGl+	HβGh+	CβG−	CβGl+	CβGh+
7	↑ 6.68 ± 0.53 ^a^	↑ 7.51 ± 0.45 ^b^	↑ 7.47 ± 0.7 ^c^	↓ 2.29 ± 1.36 *^,e^	↑ 1.67 ± 1.27 *^,f^	↑ 0.12 ± 1.30 *^,g^
21	↑ 20.63 ± 0.84 ^a^	↑ 21.83 ± 1.07 ^b^	↑ 23.43 ± 0.90 ^c,d^	↑ 13.95 ± 1.09 *^,e^	↑ 18.61 ± 1.30 ^f^	↑ 14.52 ± 2.15 *^,g,d^

^a–g^ Significant difference between groups according to the Tukey *post-hoc* test (*p* < 0.05). The same letters show statistically significant results. * Significantly different from control group (HβG−) at the same time point according to the Dunnett *post-hoc* test (*p* < 0.05). The arrows show the direction of body weight changes. ↑: increased; ↓: decreased.

**Table 2:** Biochemical values of blood plasma (mean ± SE).

Feature	Time [Days]	Healthy Group			*Colitis* Group		
		HβG−	HβGl+	HβGh+	CβG−	CβGl+	CβGh+
**ALP [U/L]**	3	47.18 ± 3.04	46.32 ± 5.34	43.48 ± 2.88	34.66 ± 2.33	39.34 ± 4.43	37.69 ± 3.92
	7	36.89 ± 2.35	38.41 ± 2.67	45.77 ± 3.04	35.43 ± 4.22	35.35 ± 2.40	35.69 ± 3.83
	21	37.1 ± 2.79	31.84 ± 3.27	36.96 ± 6.29	34.26 ± 2.98	35.93 ± 2.52	35.1 ± 3.90
**AST [U/L]**	3	43.63 ± 4.35	37.88 ± 3.87	37.43 ±1.77	40.97 ± 3.47	33.07 ± 2.18	30.72 ± 2.56
	7	39.49 ± 5.44	38.54 ± 3.45	42.52 ± 5.85	51.43 ± 3.15	41.94 ± 4.72	37.83 ± 4.38
	21	36.80 ± 2.73	37.49 ± 2.32	35.60 ± 5.69	35.26 ± 4.76	39.91 ± 5.63	40.79 ± 5.20
**ALT [U/L]**	3	16.69 ± 1.09	15.05 ± 0.75	16.41± 0.77	16.17 ± 1.27	15.74 ± 0.83	13.99± 0.81
	7	12.56 ± 0.85	13.33 ± 0.59	13.45 ± 0.71	14.95 ± 0.79	15.07 ± 1.00	12.71 ± 1.29
	21	13.80 ± 1.33	14.14 ± 1.34	15.71 ± 1.79	13.80 ± 0.48	15.94 ± 1.53	17.82 ± 1.25

The data are presented as mean ± SE. The results are no statistically significant. ALP—alkaline phosphatase; AST—aspartate aminotransferase; ALT—alanine aminotransferase.

**Table 3:** Hematological values (mean ± SE).

Feature	Time [Days]	Healthy Group			*Colitis* Group		
		HβG−	HβGl+	HβGh+	CβG−	CβGl+	CβGh+
**WBC [10^9^/L]**	3	7.71 ± 0.52	6.44 ± 0.66	5.31 ± 0.97	8.22 ± 0.91	7.16 ± 0.59	6.77 ± 0.97
	7	8.30 ± 0.66	8.30 ± 0.50	8.05 ± 0.73	8.57 ± 0.65	10.26 ± 0.69	7.87 ± 0.61
	21	7.97 ± 0.82	8.78 ± 0.56	8.59 ± 0.76	8.57 ± 0.85	8.81 ± 0.62	7.89 ± 0.26
**LYM [10^9^/L]**	3	6.36 ± 0.44	5.11 ± 0.68	3.99 ± 0.85	6.57 ± 0.83	6.29 ± 0.68	5.10 ± 0.89
	7	6.64 ± 0.56	6.76 ± 0.47	6.56 ± 0.62	6.42 ± 0.56	7.24 ± 0.38	5.67 ± 0.53
	21	6.43 ± 0.57	7.07 ± 0.50	6.77 ± 0.62	6.51 ± 0.74	7.14 ± 0.56	6.46 ± 0.24
**MID [10^9^/L]**	3	0.12 ± 0.02	0.16 ± 0.03	0.17 ± 0.04	0.17 ± 0.04	0.22 ± 0.02	0.17 ± 0.03
	7	0.17 ± 0.04	0.16 ± 0.03 ^a^	0.22 ± 0.03	0.24 ± 0.05	0.36 ± 0.04 *^,a^	0.26 ± 0.05
	21	0.12 ± 0.04	0.19 ± 0.04	0.17 ± 0.05	0.27 ± 0.03	0.15 ± 0.03	0.27 ± 0.02
**GRA [10^9^/L]**	3	1.06 ± 0.10	1.17 ± 0.09	1.06 ± 0.11	1.43 ± 0.19	1.28 ± 0.15	1.18 ± 0.17
	7	1.49 ± 0.16	1.38 ± 0.06	1.27 ± 0.14 ^a^	1.53 ± 0.15	1.87 ± 0.23	1.94 ± 0.19 ^a^
	21	1.40 ± 0.22	1.52 ± 0.17	1.58 ± 0.24	1.80 ± 0.21	1.53 ± 0.16	1.27 ± 0.13
**RBC [10^12^/L]**	3	6.63 ± 0.14	6.81± 0.10	6.53 ± 0.14 ^a,b^	6.68 ± 0.10	6.67 ± 0.11	6.72 ± 0.18
	7	7.38 ± 0.11	7.54 ± 0.18	7.44 ± 0.10 ^a^	7.37 ± 0.09	7.20 ± 0.25	7.21 ± 0.15
	21	7.07 ± 0.27	7.13 ± 0.19	7.48 ± 0.22 ^b^	7.06 ± 0.13	7.30 ± 0.12	7.22 ± 0.18
**HGB [g/dL]**	3	13.29 ± 0.14	13.59 ± 0.28	13.36 ± 0.24	13.58 ± 0.17	13.51 ± 0.17	13.60 ± 0.24
	7	13.79 ± 0.19	13.84 ± 0.18	14.11 ± 0.18	13.54 ± 0.15	13.66 ± 0.28	13.26 ± 0.27
	21	13.95 ± 0.43	13.37 ± 0.27	13.77 ± 0.29	13.44 ± 0.23	13.34 ± 0.15	13.56 ± 0.24
**HCT [%]**	3	40.66 ± 0.75	40.80 ± 0.69	40.16 ± 0.54	42.23 ± 0.47	39.83 ± 0.61	40.98 ± 0.67
	7	41.83 ± 0.45	40.45 ± 0.95	41.89 ± 0.54	41.43 ± 0.79	40.03 ± 0.85	39.67 ± 0.53
	21	41.78 ± 1.48	40.44 ± 0.74	41.98 ± 1.06	40.05 ± 0.50	39.41 ± 0.54	40.60 ± 0.66
**MCV [fl]**	3	61.25 ± 1.31	60.00 ± 0.49 ^a^	61.63 ± 1.24 ^b^	63.25 ± 1.03 ^c,d^	59.75 ± 0.92 ^e,f^	61.13 ± 1.22 ^g^
	7	56.75 ± 0.73	53.50 ± 0.91 ^a^	56.13 ± 0.67 ^b^	56.00 ± 0.80 ^c^	54.00 ± 0.65 ^e^	55.25 ± 1.39 ^g^
	21	57.33 ± 0.56	55.78 ± 0.81	56.33 ± 0.99	56.67 ± 0.62 ^d^	54.11 ± 1.33 ^f^	56.44 ± 0.96
**MCH [pg]**	3	20.08 ± 0.28	19.97 ± 0.28 ^a^	20.49 ± 0.26 ^c,d^	20.31 ± 0.28 ^e,f^	20.28 ± 0.40 ^g,h^	20.31 ± 0.50 ^i,b^
	7	18.68 ± 0.29	18.39 ± 0.28 ^a^	18.99 ± 0.25 ^c^	18.39 ± 0.18 ^e^	18.34 ± 0.22^g^	18.40 ± 0.23 ^i^
	21	18.68 ± 0.25	18.81 ± 0.31	18.47 ± 0.30 ^d^	18.77 ± 0.16 ^f^	18.29 ± 0.26^h^	18.78 ± 0.24 ^b^
**MCHC [g/dL]**	3	32.91 ± 0.33	33.30 ± 0.42	33.31 ± 0.35	32.13 ± 0.35 ^a^	34.41 ± 0.49 *^,a^	33.23± 0.34
	7	32.98 ± 0.34	34.28 ± 0.57	33.88 ± 0.61	32.23 ± 0.42	33.95 ± 0.42	33.49 ± 0.65
	21	32.58 ± 0.14	33.71 ± 0.49	32.85 ± 0.39	33.14 ± 0.36	33.43 ± 0.46	33.39 ± 0.23
**RDWc [%]**	3	16.41 ± 0.28	17.04 ± 0.20	16.98 ± 0.20	16.58 ± 0.22	17.09 ± 0.16	16.99 ± 0.31
	7	16.48 ± 0.34	17.11 ± 0.30	17.30 ± 0.30	16.79 ± 0.43	17.74 ± 0.28 *	17.44 ± 0.34
	21	16.27 ± 0.27	17.34 ± 0.30	16.93 ± 0.40	17.79 ± 0.22 *	17.98 ± 0.23 *	17.69 ± 0.29 *

WBC—white blood cells; LYM—lymphocytes; MID—monocytes, eosinophils and basophils; GRA—granulocytes; RBC—red blood cells; HGB—hemoglobin; HCT—hematocrit; MCV—mean corpuscular volume; MCH—mean corpuscular hemoglobin; MCHC—mean corpuscular hemoglobin concentration; RDWc—red blood cell distribution width; ^a–i^ Significant difference between groups according to the Tukey *post-hoc* test (*p* < 0.05). The same letters show statistically significant results within corresponding experimental group at different time points * Significantly different from control group (HβG−) at the same time point according to the Dunnett *post-hoc* test (*p* < 0.05).

## Supplementary Materials

The following are available online at https://www.mdpi.com/2076-3921/9/5/375/s1, Figure S1: The streamlined scheme of beta-glucan different fractions isolation process and their chemical structure.
