# Supplementary material for: Time-Dependent Indirect Antioxidative Effects of Oat Beta-Glucans on Peripheral Blood Parameters in the Animal Model of Colon Inflammation
Source: Antioxidants (Basel). 2020 Apr 30;9(5):375. doi: 10.3390/antiox9050375 (PMC7278816; doi:10.3390/antiox9050375)
Supplement: Supplementary file 1 [file antioxidants-09-00375-s001.pdf]

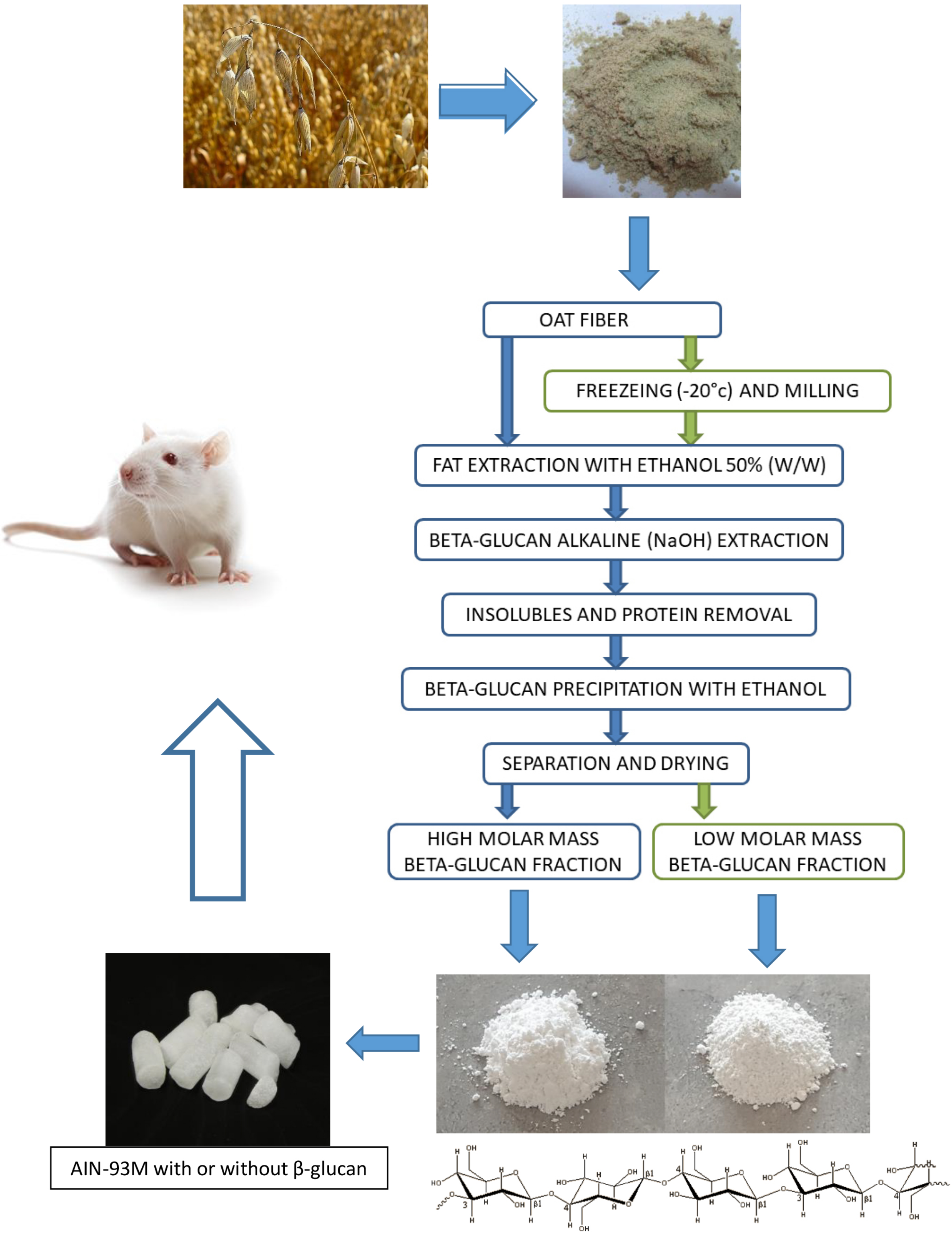

Figure S1: The streamlined scheme of beta-glucan different fractions isolation process and their chemical structure.
